# Predicting Alzheimer’s Cognitive Resilience Score: A Comparative Study of Machine Learning Models Using RNA-seq Data

**DOI:** 10.1101/2024.08.25.609610

**Published:** 2024-08-26

**Authors:** Akihiro Kitani, Yusuke Matsui

**Affiliations:** 1 Biomedical and Health Informatics Unit, Department of Integrated Health Science, Nagoya University Graduate School of Medicine, Nagoya, Japan; 2 Institute for Glyco-core Research (iGCORE), Nagoya University, 461-8673 Nagoya, Aichi, Japan.

**Keywords:** Alzheimer’s disease, machine learning, transcriptomics, Shapley additive explanations, resilience gene analyzer

## Abstract

Alzheimer’s disease (AD) is an important research topic. While amyloid plaques and neurofibrillary tangles are hallmark pathological features of AD, cognitive resilience (CR) is a phenomenon where cognitive function remains preserved despite the presence of these pathological features. This study aimed to construct and compare predictive machine learning models for CR scores using RNA-seq data from the Religious Orders Study and Memory and Aging Project (ROSMAP) and Mount Sinai Brain Bank (MSBB) cohorts. We evaluated support vector regression (SVR), random forest, XGBoost, linear, and transformer-based models. The SVR model exhibited the best performance, with contributing genes identified using Shapley additive explanations (SHAP) scores, providing insights into biological pathways associated with CR. Finally, we developed a tool called the resilience gene analyzer (REGA), which visualizes SHAP scores to interpret the contributions of individual genes to CR. REGA is available at https://igcore.cloud/GerOmics/REsilienceGeneAnalyzer/.

## Introduction

Alzheimer’s disease (AD) remains a critical area of research because of its largely unknown causes and pathophysiology, as well as the urgent need for effective treatments. In AD, cognitive resilience (CR) refers to the ability of some individuals to maintain cognitive function despite the presence of amyloid plaques and neurofibrillary tangles. These plaques and tangles are the hallmark pathological features of the disease. CR is defined as the discrepancy between an individual’s observed and expected cognitive functions based on the extent of brain pathology [1–3]. Several factors have been associated with CR, including sex/gender differences [4], educational level, and brain weight [5,6]. Studies have also identified other factors, such as personality traits, Parkinson’s disease, depression, life activities, and eudaimonic well-being, to CR [7–9]. Despite these findings, the molecular mechanisms underlying CR remain unclear.

Omics analysis, which comprehensively examines molecules within an organism, has been widely used in AD research to analyze pathologies, elucidate drug mechanisms, and discover biomarkers [10]. Several studies have utilized omics in their investigations on CR [11–14]. Genomics studies have reported associations with genes such as APOE, BDNF, Klotho, SNX25, PDLIM3, SORBS2, CD44, NPHP1, CADPS2, and GREM2, with CR [15–22]. Transcriptomic and proteomic studies have suggested several molecular mechanisms, including the association of proteins such as NRN1, ACTN4, EPHX4, RPH3A, SGTB, CPLX1, SH3GL1, and UBA1, with CR [23–30].

However, commonly used statistical methods in omics studies frequently fail to detect significant factors or provide detailed mechanistic insights into CR. Machine learning algorithms have been applied to large and complex datasets such as RNA-seq data [31,32]. These algorithms have been successful in identifying disease-related gene signatures, predicting prognosis, stratifying patients, and facilitating the classification of disease subtypes [33–38]. For example, Ahammad et al. developed an AD prediction tool called AITeQ, which utilizes machine learning models and RNA-seq data to identify a set of genes with high accuracy [38]. While machine learning has been successfully applied to AD prediction, few studies have focused on utilizing these techniques to analyze omics data, particularly for CR.

In this study, we aimed to analyze the pathology of CR in AD by constructing regression models. These models predict CR scores using RNA-seq data obtained from the ROSMAP [39] and MSBB [40] cohorts. For both datasets, the support vector machine (SVM) model exhibited the best performance. In addition, we used Shapley additive explanations (SHAP) [41] scores to identify genes contributing to the model’s predictions, revealing contributions from genes associated with energy metabolism pathways. Finally, we developed a resilience gene analyzer (REGA), which visualizes the SHAP scores calculated by the best-performing SVR model. This study lays the foundation for applying machine learning to cognitive resilience research in AD and paves the way for experimental validation of the findings.

## Materials and methods

**Fig. 1.**
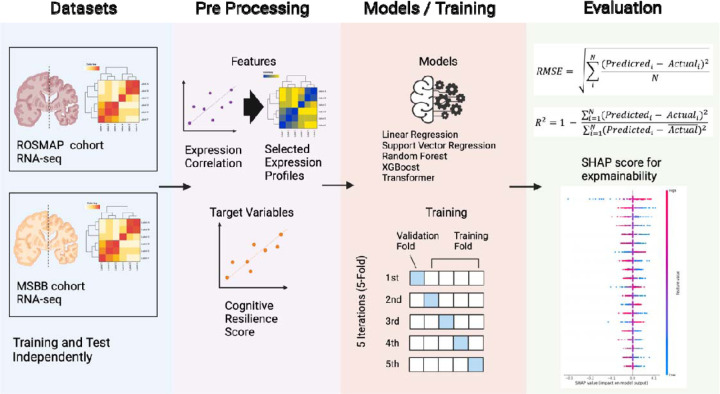
Overview of the data analysis performed in this study. RNA-seq datasets from the ROSMAP [39] and MSBB [40] cohorts were analyzed independently. The target variable was the resilience score, and the features were the expression levels of genes that were highly correlated with the resilience score. The models used in the present work included linear models, SVR [43], random forest [44], XGBoost [45], and transformer-based models [46,47]. Learning was performed using 5-fold cross-validation. The evaluation metrics were RMSE and R^2^, and gene contributions were calculated using the SHAP scores [41] in the best-performing model.

Fig. 1 shows the analytical workflow used in this study. The detailed flow from data collection and pre-processing to training is shown in Supplementary Fig. S1.

### Data Collection and Preprocessing

To calculate the CR score, we used RNA-seq data from the MSBB and ROSMAP cohorts, which included both cognitive function and pathological assessments related to amyloid-β and tau. We used conditional quantile-normalized data from these cohorts (MSBB: https://doi.org/10.7303/syn2580853; ROSMAP: https://doi.org/10.7303/syn2580853). The details of sample collection and data processing have been described previously [39,40]. Outliers identified using principal component analysis were excluded. In the ROSMAP cohort, the tissues included the dorsolateral prefrontal cortex, head of the caudate nucleus, and posterior cingulate cortex. The head of the caudate nucleus and sequencing batch number 9 were excluded from the analysis. The MSBB cohort included tissue from the frontal pole, inferior frontal gyrus, parahippocampal gyrus, prefrontal cortex, and superior temporal gyrus. Finally, we utilized 1247 samples from the MSBB cohort and 1549 samples from the ROSMAP cohort, including patients with dementia and healthy controls. The batch effects were corrected using ComBat [42] based on the batch information of each dataset.

### Quantification of CR

A linear regression analysis was performed to calculate CR scores. The score for each individual was defined as the difference between observed and predicted cognitive functions. To predict cognitive function, clinical dementia rating (CDR) was used as the target variable for the MSBB cohort and the mini-mental state examination (MMSE) was used for the ROSMAP cohort. The explanatory variables included the CERAD score and Braak stage.

### Feature Selection

In this study, features with high Pearson correlation coefficients and CR scores were selected for analysis using feature sets of 1000, 2000, 3000, 4000, and 5000. For example, in the case of a 1000-feature set, the top 500 features with positive correlations and the top 500 features with negative correlations were selected. The features were normalized using the MinMaxScaler function from the scikit-learn library in Python.

### Machine Learning Models

#### Linear Regression Model

Linear regression is a basic statistical method that is used to model the relationship between a dependent variable and one or more independent variables. In this study, we implemented a linear regression model using the PyTorch library in Python. The model was trained using the Adam optimizer and the mean squared error (MSE) loss function. The hyperparameter settings included a learning rate of 0.01, 300 epochs, and a batch size of 16.

#### Support Vector Regression

Support vector regression (SVR) [43] is a type of SVM used for regression tasks. SVR aims to determine a function that approximates the target values with minimal deviation by fitting a hyperplane within a margin of tolerance to the data points. The SVR is robust against outliers and can be used to model nonlinear relationships using kernel functions. This method is particularly useful for high-dimensional complex data. The SVR function from the scikit-learn library was used.

#### Random Forest

Random forest [44] is an ensemble learning method that constructs multiple decision trees during training and outputs the average predictions of individual trees. This approach improves the prediction accuracy and controls overfitting by averaging the results of multiple trees. Each tree was trained on a random subset of the data, introducing diversity and reducing variance. Random forests can handle many features and capture intricate data patterns. In this study, we used the RandomForestRegressor function from the scikit-learn library.

#### Extreme gradient boosting

Extreme gradient boosting (XGBoost) [45] is an advanced implementation of gradient boosting designed for speed and performance. It builds an ensemble of trees sequentially, with each new tree correcting the errors of the predecessor. XGBoost uses regularization techniques to prevent overfitting and incorporates various optimization algorithms to improve the training efficiency. We used the XGBRegressor from the XGBoost library.

#### Transformer-based Model

Initially developed for natural language processing tasks [46], transformer-based models have shown great promise in various domains, including RNA-seq [14,47]. These models utilize self-attention mechanisms to weigh the importance of different input features, making them highly effective for modeling complex relationships. In this study, we used a recently reported transformer-based model [47] with some modifications to predict CR by leveraging its ability to learn intricate patterns from high-dimensional RNA-seq data. The architecture of our transformer-based model includes two attention layers, each with four multihead attention mechanisms. We used the GELU activation function and included dropout layers at a rate of 0.4 to prevent overfitting. The feedforward network within the transformer model has a hidden layer dimensionality of 256. The model was optimized using the Adam optimizer with a learning rate of 0.0001. Additionally, the model was trained for 500 epochs with a batch size of 16.

### K-fold Cross Validation and Hyperparameter Tuning

To ensure the robustness and generalizability of the predictive models, k-fold cross-validation and hyperparameter tuning were implemented. We utilized the scikit-learn library to perform grid-search cross-validation and systematically evaluated different hyperparameter combinations to identify the optimal configuration for each model. We employed a nested cross-validation approach with an outer loop of 5-fold cross-validation to assess generalization performance. An inner loop of 5-fold cross-validation was used within the training set to perform a grid-search for hyperparameter tuning. For each fold in the outer cross-validation, the data were split into training and test sets. Within the training set, grid-search cross-validation was performed using the defined hyperparameter grid. The best hyperparameters were selected based on the lowest MSE from the inner loop. The best model was retrained using the entire training set and evaluated using the test set. Performance metrics, including R^2^ and the root mean squared error (RMSE), were calculated for both the training and test sets. The average performance metrics across all folds were computed to provide an overall assessment of the model performance. This systematic approach allowed us to thoroughly evaluate and optimize our models, ensuring reliable and accurate predictions of CR. Detailed results, including the hyperparameters and performance metrics for each fold, are provided in Supplementary Tables S2 and S4.

### Explaining Predictions Using SHAP

Using the SHAP library, we visualized the genes that contributed to the predictions made by the best models. For the SVM model, which demonstrated the best performance in terms of R^2^ across all rounds of cross-validation for each dataset, the SHAP scores were calculated using 100 test samples with the KernelExplainer function. The results were plotted using the summaryplot function.

### Enrichment Analysis for Top SHAP Score Genes

Enrichment analysis was performed to explore the biological functions of the target gene set. The gene ontology biological process (GObp), Hallmark, KEGG pathway, and Reactome datasets (version 2023.1) were downloaded from the Molecular Signatures Database (MsigDB) (https://www.gsea-msigdb.org/gsea/msigdb). For the analysis, the enrichment function in the clusterProfiler package in R was used, and p-values <0.05, after Benjamini–Hochberg (BH) correction, were considered significant.

### Establishment of REGA

Using the R Shiny app, we developed a tool named REGA to visualize the SHAP score output from the MSBB and ROSMAP cohorts. By selecting a dataset and genes, users can observe the distribution of SHAP scores for the selected genes and plot the SHAP scores for individual samples, indicating the contribution of the selected genes to resilience score predictions.

## Results

### Superior Performance of SVR in Predicting Resilience Score in the MSBB Cohort

**Fig. 2.**
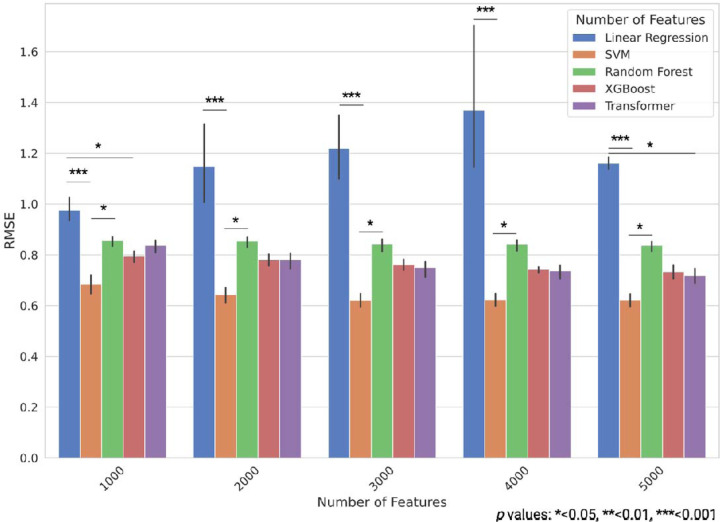
Prediction performances of different machine learning models using various feature sets in the MSBB study data. RMSE for each model is shown for the test data, with error bars representing standard error. Statistical significance was assessed using the Kruskal–Wallis test followed by Dunn’s multiple comparison test, with p-values adjusted by Bonferroni correction: *<0.05, **<0.01, ***<0.001; n = 5 for 5-fold cross-validation).

First, we constructed models to predict resilience scores using the MSBB cohort and compared their prediction accuracies on the test dataset. We selected features based on the top 1000, 2000, 3000, 4000, and 5000 Pearson correlation coefficients with the resilience score. For training, we used 5-fold cross-validation and evaluated the models based on the average RMSE and R^2^ values. The SVR model exhibited the best performance across all the feature sets. In particular, the SVR model with 3000 features exhibited the best performance, with an average RMSE of 0.621 (standard error, SE 0.0137) and an average R^2^ of 0.614 (SE 0.0137) (Fig. 2, Supplementary Fig. S2, and Supplementary Tables S1 and S2). Following the SVR model, the transformer, XGBoost, and random forest models showed progressively lower performance. The Linear model exhibited the lowest prediction accuracy. In addition, no significant differences were observed in the results based on the number of features used.

### Superior Performance of SVR and Transformer Models in the ROSMAP Cohort

**Fig. 3.**
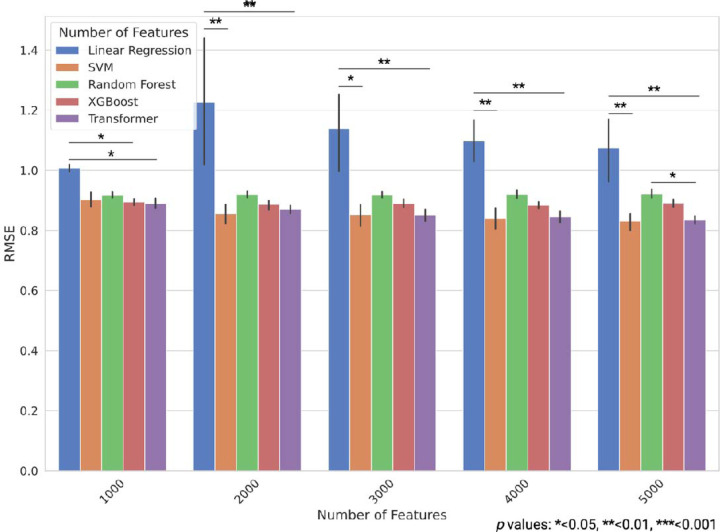
Prediction performances of different machine learning models using various feature sets in the ROSMAP study data. RMSE for each model is shown for the test data, with error bars representing standard error. Statistical significance was assessed using the Kruskal–Wallis test followed by the Dunn’s multiple comparison test, with p-values adjusted by Bonferroni correction: *<0.05, **<0.01, ***<0.001; n = 5 for 5-fold cross-validation).

We performed a similar analysis using the ROSMAP cohort data. In the ROSMAP cohort, the transformer model exhibited the best performance with 1000 and 3000 features, whereas the SVR model exhibited the best performance with 2000, 4000, and 5000 features. The SVR model consistently exhibited the best performance using 5000 features. This model achieved an average RMSE of 0.831 (SE 0.0141) and an average R^2^ of 0.302 (SE 0.0252) (Fig. 3, Supplementary Fig. S3, Supplementary Tables S3 and S4).

### Explanation of the Best Models Through SHAP Scores

**Fig. 4.**
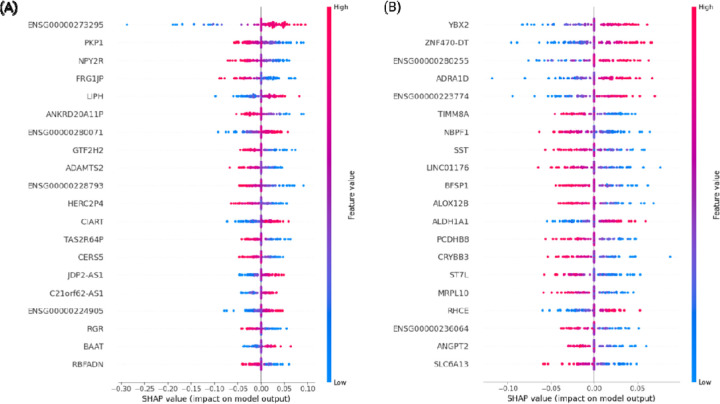
SHAP values of the best models for predicting resilience scores in the (A) MSBB and (B) ROSMAP cohorts. SHAP values were calculated for 100 data points, with each dot representing an individual’s patient’s data.

In the MSBB and ROSMAP datasets, the SVR model exhibited the best performance (R^2^ = 0.649 with 3000 features in the MSBB dataset and R^2^ = 0.364 with 4000 features in the ROSMAP dataset) (Supplementary Tables S2 and S4). To identify the genes contributing to model predictions, SHAP values were calculated for the best model in each dataset using 100 test samples (Fig. 4). The SHAP values indicate the extent to which each feature (gene) contributes to the output of the model. SHAP scores are based on cooperative game theory, which considers all possible combinations of features to fairly distribute the “payout” among them [41]. Genes such as PKP1, NPY2R, FRG1JP, and ADAMTS2 were identified in the MSBB cohort, whereas YBX2, ADRA1D, TIMM8A, ANGPT2, and SLC6A13 were identified in the ROSMAP cohort.

### Enrichment Analysis of Top SHAP Score Genes

The top 500 SHAP score genes from each dataset were extracted, and common genes were identified, resulting in 39 common genes (Supplemengary Fig. S4A). Enrichment analysis was performed for each dataset. The 39 common genes between the two cohorts showed significant enrichment in the ATP metabolic process and amino sugar catabolic process pathways with a BH-adjusted p-value of less than 0.05 (each 0.0478) (Supplementary Fig. S4B and Table 1).

### Establishment of REGA

Finally, using the R Shiny application, we developed a tool to visualize the SHAP scores calculated from the best SVR models in both the MSBB and ROSMAP cohorts. This tool is accessible at https://igcore.cloud/GerOmics/REsilienceGeneAnalyzer/.

## Discussion

In this study, we constructed predictive models for AD resilience scores using RNA-seq data and machine learning techniques. Our results showed that compared to the random forest, XGBoost, linear, and transformer-based models, the SVR model had the best performance in the MSBB cohort, whereas both the SVR and transformer-based models exhibited the best performance in the ROSMAP cohort.

Traditional statistical methods in AD resilience research often face challenges, such as low correlation and difficulty in detecting variable genes, particularly in CR research. These approaches may not identify all relevant molecules or capture the nuanced effects of resilience, particularly given the significant differences between pathological and normal conditions. In our study, the machine learning SVR model achieved an R^2^ of 0.649 in the MSBB cohort, demonstrating a relatively high accuracy for the regression models. Nevertheless, the prediction accuracy in the ROSMAP cohort was lower, possibly because of differences in RNA-seq quality, batch effects, and variability in resilience scores. Future studies should focus on expanding the datasets to include more accurate clinical and resilience scores, thereby improving model robustness and prediction accuracy. Transformer-based models, which have recently been reported to achieve high accuracy in cancer classification studies [47], have ranked second in this study. This outcome can be attributed to the sample size constraints. This study used data from 1247 samples in the MSBB cohort and 1549 samples in the ROSMAP cohort, whereas the previous study used data from 10,340 and 713 samples for 33 cancer types and 23 normal tissues, respectively. Transformer-based models may require larger datasets to effectively learn complex relationships within the data. However, the transformer model exhibited the best performance in several cases in the ROSMAP cohort with a larger sample size.

SHAP [41] analysis identified genes contributing to the resilience score predictions in the best-performing models for both the MSBB and ROSMAP cohorts. Many of these genes have been previously associated with AD [48–55]. For example, somatostatin (SST) has been linked to abnormal levels of neuropeptides, impaired function, and hyperactivity of SST-positive interneurons (SST-IN) co-localized with amyloid-β plaques in AD [49,50]. Recent single-nucleus RNA-seq studies have reported that SST neurons are associated with cognitive resilience [12]. Additionally, enrichment analysis of 39 common genes revealed enrichment in ATP metabolic and amino sugar catabolic process pathways. Previous studies have also reported that amino acid metabolism pathways are associated with CR [21]. Additionally, amyloid positivity without cognitive impairment was associated with the preservation of youthful brain aerobic glycolysis [56].

Furthermore, we developed an SVR-based resilience score prediction tool called REGA. The SVR model provided the best results for both the ROSMAP and MSBB cohorts. This tool allows users to analyze how well the SVM explains resilience based on one or more genes detected in either cohort. It is anticipated that this tool will provide valuable reference data for analyses and experimental validation across different datasets.

This study has several limitations, with sample size being a critical factor. Recently, transformer-based models have shown high prediction accuracy in various cases compared to existing machine learning models. However, because of their complexity, transformers require large sample sizes to achieve high accuracy. In this study, the limited sample size led to the identification of SVM as the best model. Reconstructing a model with a larger sample size may reveal different models with better accuracy. Increasing the sample size is expected to enhance the overall prediction accuracy. Second, the robust measurement of resilience scores is challenging. Although the same explanatory variables (Braak stage and CERAD score) were used in both the ROSMAP and MSBB cohorts, different target variables (MMSE and CDR scores) were used to construct the linear models and calculate the resilience scores. This methodological difference may be a reason for the lower prediction accuracy of the ROSMAP cohort. Different methods of calculating the resilience scores prevented data integration and cross-validation of the models between cohorts. Finally, biological and technical variations in RNA-seq data, such as batch effects, sequencing depth, and normalization methods, can introduce variability. Machine learning models are susceptible to these factors; therefore, appropriate data preprocessing and normalization methods are essential to ensure accurate classification.

## Conclusion

Our study demonstrated that machine learning models, particularly SVR, can effectively predict cognitive resilience scores in AD using RNA-seq data. Despite some variability in prediction accuracy across different cohorts, integrating more comprehensive datasets and improving resilience scoring methods could further enhance model performance. Additionally, these advancements may provide deeper insights into the molecular mechanisms underlying cognitive resilience.

## Supplementary Material

Supplement 2

Supplement 3

Supplement 4

Supplement 5

1

## Figures and Tables

**Fig. 5.**
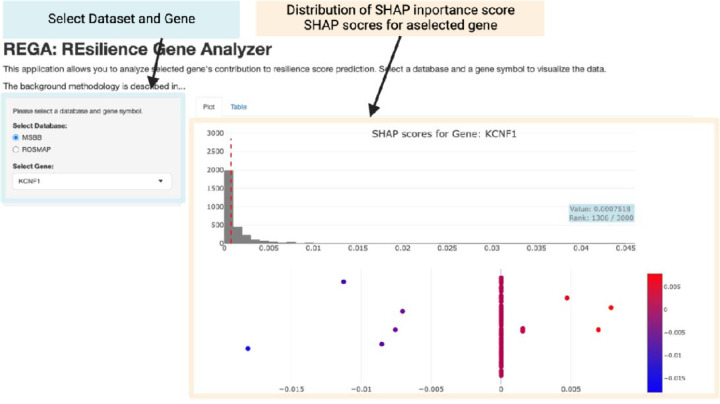
Visualization of gene contribution to the prediction of resilience scores. The REGA tool interface for visualizing the contribution of individual genes to resilience score predictions is shown. This tool allows users to select a dataset (MSBB or ROSMAP) and specific genes for the analysis. The left panel shows the selection options for datasets and genes. The top-right panel shows the distribution of SHAP scores for all genes, with a dashed line indicating the importance score for the selected gene. The bottom-right panel shows the SHAP scores for individual samples, indicating the contribution of the selected genes to the resilience score predictions.

**Table 1.** Top 10 pathways from the GOBP, Reactome, Hallmark, and KEGG databases based on q-values for the combined dataset, as well as for the MSBB and ROSMAP cohorts individually.

Combined	pvalue	p.adjust	qvalue	geneset
GOBP_REGULATION_OF_ATP_METABOLIC_PROCESS	0.0002	0.0478	0.0394	GObp
GOBP_AMINO_SUGAR_CATABOLIC_PROCESS	0.0002	0.0478	0.0394	GObp
GOBP_REGULATION_OF_NUCLEOTIDE_METABOLIC_PROCESS	0.0004	0.0525	0.0433	GObp
REACTOME_RHOJ_GTPASE_CYCLE	0.0035	0.1757	0.1406	Reactome
REACTOME_SYNTHESIS_OF_SUBSTRATES_IN_N_GLYCAN_BIOSYTHESIS	0.0046	0.1757	0.1406	Reactome
REACTOME_BIOSYNTHESIS_OF_THE_N_GLYCAN_PRECURSOR_DOLICHOL_LIPID_LINKED_OLIGOSACCHARIDE_LLO_AND_TRANSFER_TO_A_NASCENT_PROTEIN	0.0070	0.1757	0.1406	Reactome
REACTOME_METABOLISM_OF_CARBOHYDRATES	0.0112	0.1757	0.1406	Reactome
REACTOME_ACTIVATION_OF_PPARGC1A_PGC_1ALPHA_BY_PHOSPHORYLATION	0.0162	0.1757	0.1406	Reactome
REACTOME_CASPASE_MEDIATED_CLEAVAGE_OF_CYTOSKELETAL_PROTEINS	0.0194	0.1757	0.1406	Reactome
REACTOME_ETHANOL_OXIDATION	0.0194	0.1757	0.1406	Reactome
MSBB				
GOBP_NEGATIVE_REGULATION_OF_CAMP_MEDIATED_SIGNALING	0.0000	0.1073	0.1034	GObp
GOBP_EXTERNAL_ENCAPSULATING_STRUCTURE_ORGANIZATION	0.0001	0.1680	0.1619	GObp
GOBP_MYOBLAST_FUSION	0.0003	0.2585	0.2492	GObp
GOBP_HEPATOCYTE_APOPTOTIC_PROCESS	0.0005	0.3357	0.3237	GObp
GOBP_REGULATION_OF_MYOBLAST_FUSION	0.0006	0.3357	0.3237	GObp
GOBP_RESPONSE_TO_TEMPERATURE_STIMULUS	0.0007	0.3530	0.3403	GObp
GOBP_NEGATIVE_REGULATION_OF_CELL_CYCLE_PROCESS	0.0010	0.3662	0.3530	GObp
GOBP_COLLAGEN_FIBRIL_ORGANIZATION	0.0012	0.3662	0.3530	GObp
GOBP_IMMATURE_B_CELL_DIFFERENTIATION	0.0013	0.3662	0.3530	GObp
GOBP_MITOTIC_CELL_CYCLE_PHASE_TRANSITION	0.0013	0.3662	0.3530	GObp
ROSMAP				
REACTOME_RHOB_GTPASE_CYCLE	0.0004	0.1249	0.1202	Reactome
REACTOME_RHOC_GTPASE_CYCLE	0.0006	0.1249	0.1202	Reactome
REACTOME_RHOA_GTPASE_CYCLE	0.0006	0.1249	0.1202	Reactome
HALLMARK_HYPOXIA	0.0054	0.2434	0.2334	Hallmark
KEGG_BIOSYNTHESIS_OF_UNSATURATED_FATTY_ACIDS	0.0058	0.3136	0.3136	KEGG
KEGG_NUCLEOTIDE_EXCISION_REPAIR	0.0063	0.3136	0.3136	KEGG
REACTOME_RHO_GTPASE_CYCLE	0.0028	0.3771	0.3632	Reactome
REACTOME_CDC42_GTPASE_CYCLE	0.0031	0.3771	0.3632	Reactome
REACTOME_RHOJ_GTPASE_CYCLE	0.0041	0.3792	0.3652	Reactome
REACTOME_CROSSLINKING_OF_COLLAGEN_FIBRILS	0.0047	0.3792	0.3652	Reactome

## Data Availability

All the data used in this study are publicly accessible. RNA-seq data were accessed using the AD Knowledge Portal (https://doi.org/10.7303/syn2580853; ROSMAP: https://doi.org/10.7303/syn2580853).

## Key Points

We developed predictive models for Alzheimer’s disease resilience scores and compared their prediction accuracies with that of the support vector machine model, which demonstrated the highest accuracy. The prediction accuracy was lower in the ROSMAP cohort than in the MSBB cohort. This discrepancy may be attributed to differences in RNA-seq data quality, batch effects, and the robustness of the resilience scores. Therefore, larger and more robust datasets with accurate cognitive function measurements are required. We developed a user-friendly tool, resilience gene analyzer (REGA), which provides straightforward access to the analysis results from this study.

## References

[1] de Vries LE, Huitinga I, Kessels HW, The concept of resilience to Alzheimer’s Disease: current definitions and cellular and molecular mechanisms. Mol. Neurodegener. 2024; 19:33 38589893 10.1186/s13024-024-00719-7 PMC11003087

[2] Negro D, Opazo P. Cognitive resilience in Alzheimer’s disease: from large-scale brain networks to synapses. Brain Commun. 2024; 6:fcae050 38425748 10.1093/braincomms/fcae050 PMC10903981

[3] Arenaza-Urquijo EM, Vemuri P. Resistance vs resilience to Alzheimer disease: Clarifying terminology for preclinical studies. Neurology 2018; 90:695–703 29592885 10.1212/WNL.0000000000005303 PMC5894932

[4] Arenaza-Urquijo EM, Boyle R, Casaletto K, Sex and gender differences in cognitive resilience to aging and Alzheimer’s disease. Alzheimers. Dement. 2024; 10.1002/alz.13844 PMC11350140 38967222

[5] Aiello Bowles EJ, Crane PK, Walker RL, Cognitive resilience to Alzheimer’s disease pathology in the human brain. J. Alzheimers. Dis. 2019; 68:1071–1083 30909217 10.3233/JAD-180942 PMC8357030

[6] Ossenkoppele R, Lyoo CH, Jester-Broms J, Assessment of demographic, genetic, and imaging variables associated with brain resilience and cognitive resilience to pathological tau in patients with Alzheimer disease. JAMA Neurol. 2020; 77:632–642 32091549 10.1001/jamaneurol.2019.5154 PMC7042808

[7] Willroth EC, James BD, Graham EK, Well-being and cognitive resilience to dementia-related neuropathology. Psychol. Sci. 2023; 34:283–297 36473124 10.1177/09567976221119828 PMC10068507

[8] Yao T, Sweeney E, Nagorski J, Quantifying cognitive resilience in Alzheimer’s Disease: The Alzheimer’s Disease Cognitive Resilience Score. PLoS One 2020; 15:e0241707 33152028 10.1371/journal.pone.0241707 PMC7643963

[9] Bocancea DI, Svenningsson AL, van Loenhoud AC, Determinants of cognitive and brain resilience to tau pathology: a longitudinal analysis. Brain 2023; 146:3719–3734 36967222 10.1093/brain/awad100 PMC10473572

[10] Tan MS, Cheah P-L, Chin A-V, A review on omics-based biomarkers discovery for Alzheimer’s disease from the bioinformatics perspectives: Statistical approach vs machine learning approach. Comput. Biol. Med. 2021; 139:104947 34678481 10.1016/j.compbiomed.2021.104947

[11] Neuner SM, Telpoukhovskaia M, Menon V, Translational approaches to understanding resilience to Alzheimer’s disease. Trends Neurosci. 2022; 45:369–383 35307206 10.1016/j.tins.2022.02.005 PMC9035083

[12] Mathys H, Peng Z, Boix CA, Single-cell atlas reveals correlates of high cognitive function, dementia, and resilience to Alzheimer’s disease pathology. Cell 2023; 186:4365–4385.e27 37774677 10.1016/j.cell.2023.08.039 PMC10601493

[13] Mathys H, Boix CA, Akay LA, Single-cell multiregion dissection of Alzheimer’s disease. Nature 2024; 1–11 10.1038/s41586-024-07606-7 PMC11338834 39048816

[14] Berson E, Sreenivas A, Phongpreecha T, Whole genome deconvolution unveils Alzheimer’s resilient epigenetic signature. Nat. Commun. 2023; 14:4947 37587197 10.1038/s41467-023-40611-4 PMC10432546

[15] Lopera F, Marino C, Chandrahas AS, Resilience to autosomal dominant Alzheimer’s disease in a Reelin-COLBOS heterozygous man. Nat. Med. 2023; 29:1243–1252 37188781 10.1038/s41591-023-02318-3 PMC10202812

[16] Phongpreecha T, Godrich D, Berson E, Quantitative estimate of cognitive resilience and its medical and genetic associations. Alzheimers. Res. Ther. 2023; 15:192 37926851 10.1186/s13195-023-01329-z PMC10626669

[17] Arboleda-Velasquez JF, Lopera F, O’Hare M, Resistance to autosomal dominant Alzheimer’s disease in an APOE3 Christchurch homozygote: a case report. Nat. Med. 2019; 25:1680–1683 31686034 10.1038/s41591-019-0611-3 PMC6898984

[18] Franzmeier N, Ren J, Damm A, The BDNFVal66Met SNP modulates the association between beta-amyloid and hippocampal disconnection in Alzheimer’s disease. Mol. Psychiatry 2021; 26:614–628 30899092 10.1038/s41380-019-0404-6 PMC6754794

[19] Vélez JI, Chandrasekharappa SC, Henao E, Pooling/bootstrap-based GWAS (pbGWAS) identifies new loci modifying the age of onset in PSEN1 p.Glu280Ala Alzheimer’s disease. Mol. Psychiatry 2013; 18:568–575 22710270 10.1038/mp.2012.81 PMC3596442

[20] Ramanan VK, Lesnick TG, Przybelski SA, Coping with brain amyloid: genetic heterogeneity and cognitive resilience to Alzheimer’s pathophysiology. Acta Neuropathol. Commun. 2021; 9:48 33757599 10.1186/s40478-021-01154-1 PMC7986461

[21] Dumitrescu L, Mahoney ER, Mukherjee S, Genetic variants and functional pathways associated with resilience to Alzheimer’s disease. Brain 2020; 143:2561–2575 32844198 10.1093/brain/awaa209 PMC7447518

[22] Belloy ME, Napolioni V, Han SS, Association of klotho-VS heterozygosity with risk of Alzheimer disease in individuals who carry APOE4. JAMA Neurol. 2020; 77:849–862 32282020 10.1001/jamaneurol.2020.0414 PMC7154955

[23] Yu L, Tasaki S, Schneider JA, Cortical proteins associated with cognitive resilience in community-dwelling older persons. JAMA Psychiatry 2020; 77:1172–1180 32609320 10.1001/jamapsychiatry.2020.1807 PMC7330835

[24] Carlyle BC, Kandigian SE, Kreuzer J, Synaptic proteins associated with cognitive performance and neuropathology in older humans revealed by multiplexed fractionated proteomics. Neurobiol. Aging 2021; 105:99–114 34052751 10.1016/j.neurobiolaging.2021.04.012 PMC8338777

[25] Lu T, Aron L, Zullo J, REST and stress resistance in ageing and Alzheimer’s disease. Nature 2014; 507:448–454 24670762 10.1038/nature13163 PMC4110979

[26] Barker SJ, Raju RM, Milman NEP, MEF2 is a key regulator of cognitive potential and confers resilience to neurodegeneration. Sci. Transl. Med. 2021; 13:eabd7695 34731014 10.1126/scitranslmed.abd7695 PMC9258338

[27] Barroeta-Espar I, Weinstock LD, Perez-Nievas BG, Distinct cytokine profiles in human brains resilient to Alzheimer’s pathology. Neurobiol. Dis. 2019; 121:327–337 30336198 10.1016/j.nbd.2018.10.009 PMC6437670

[28] Bartosch AMW, Youth EHH, Hansen S, ZCCHC17 modulates neuronal RNA splicing and supports cognitive resilience in Alzheimer’s disease. bioRxivorg 2023; 10.1523/JNEUROSCI.2324-22.2023 PMC10860597 38050142

[29] Huang Z, Merrihew GE, Larson EB, Brain proteomic analysis implicates actin filament processes and injury response in resilience to Alzheimer’s disease. Nat. Commun. 2023; 14:2747 37173305 10.1038/s41467-023-38376-x PMC10182086

[30] Yu L, Petyuk VA, Gaiteri C, Targeted brain proteomics uncover multiple pathways to Alzheimer’s dementia. Ann. Neurol. 2018; 84:78–88 29908079 10.1002/ana.25266 PMC6119500

[31] Pandey D, Onkara Perumal P. A scoping review on deep learning for next-generation RNA-Seq. data analysis. Funct. Integr. Genomics 2023; 23:134 37084004 10.1007/s10142-023-01064-6

[32] Deshpande D, Chhugani K, Chang Y, RNA-seq data science: From raw data to effective interpretation. Front. Genet. 2023; 14:10.3389/fgene.2023.997383 PMC10043755 36999049

[33] Al Olaimat M, Martinez J, Saeed F, PPAD: A deep learning architecture to predict progression of Alzheimer’s disease. bioRxivorg 2023; 10.1093/bioinformatics/btad249 PMC10311312 37387135

[34] Eteleeb AM, Novotny BC, Tarraga CS, Brain high-throughput multi-omics data reveal molecular heterogeneity in Alzheimer’s disease. PLoS Biol. 2024; 22:e3002607 38687811 10.1371/journal.pbio.3002607 PMC11086901

[35] Park C, Ha J, Park S. Prediction of Alzheimer’s disease based on deep neural network by integrating gene expression and DNA methylation dataset. Expert Syst. Appl. 2020; 140:112873

[36] Beebe-Wang N, Celik S, Weinberger E, Unified AI framework to uncover deep interrelationships between gene expression and Alzheimer’s disease neuropathologies. Nat. Commun. 2021; 12:5369 34508095 10.1038/s41467-021-25680-7 PMC8433314

[37] Rodriguez S, Hug C, Todorov P, Machine learning identifies candidates for drug repurposing in Alzheimer’s disease. Nat. Commun. 2021; 12:1033 33589615 10.1038/s41467-021-21330-0 PMC7884393

[38] Ahammad I, Lamisa AB, Bhattacharjee A, AITeQ: a machine learning framework for Alzheimer’s prediction using a distinctive five-gene signature. Brief. Bioinform. 2024; 25:10.1093/bib/bbae291 PMC11179120 38877887

[39] De Jager PL, Ma Y, McCabe C, A multi-omic atlas of the human frontal cortex for aging and Alzheimer’s disease research. Sci. Data 2018; 5:180142 30084846 10.1038/sdata.2018.142 PMC6080491

[40] Wang M, Beckmann ND, Roussos P, The Mount Sinai cohort of large-scale genomic, transcriptomic and proteomic data in Alzheimer’s disease. Sci. Data 2018; 5:180185 30204156 10.1038/sdata.2018.185 PMC6132187

[41] Lundberg SM, Lee S-I. A unified approach to interpreting model predictions. Neural Inf Process Syst 2017; 4765–4774

[42] Johnson WE, Li C, Rabinovic A. Adjusting batch effects in microarray expression data using empirical Bayes methods. Biostatistics 2007; 8:118–127 16632515 10.1093/biostatistics/kxj037

[43] Drucker H, Burges C, Kaufman L, Support Vector Regression Machines. Neural Inf Process Syst 1996; 9:155–161

[44] Breiman L. Random Forests | Machine Learning. Mach. Learn. 2001; 45:5–32

[45] Chen T, Guestrin C. XGBoost: A Scalable Tree Boosting System. Proceedings of the 22nd ACM SIGKDD International Conference on Knowledge Discovery and Data Mining 2016;

[46] Vaswani A, Shazeer N, Parmar N, Attention is all you need. arXiv [cs.CL] 2017;

[47] Zhang T-H, Hasib MM, Chiu Y-C, Transformer for Gene Expression Modeling (T-GEM): An interpretable Deep Learning model for gene expression-based phenotype predictions. Cancers (Basel) 2022; 14:4763 36230685 10.3390/cancers14194763 PMC9562172

[48] Pain S, Brot S, Gaillard A. Neuroprotective effects of neuropeptide Y against neurodegenerative disease. Curr. Neuropharmacol. 2022; 20:1717–1725 34488599 10.2174/1570159X19666210906120302 PMC9881060

[49] Saito T, Iwata N, Tsubuki S, Somatostatin regulates brain amyloid beta peptide Abeta42 through modulation of proteolytic degradation. Nat. Med. 2005; 11:434–439 15778722 10.1038/nm1206

[50] Almeida VN. Somatostatin and the pathophysiology of Alzheimer’s disease. Ageing Res. Rev. 2024; 96:102270 38484981 10.1016/j.arr.2024.102270

[51] Balmorez T, Sakazaki A, Murakami S. Genetic networks of Alzheimer’s disease, aging, and longevity in humans. Int. J. Mol. Sci. 2023; 24:10.3390/ijms24065178 PMC10049434 36982253

[52] Yamakage Y, Kato M, Hongo A, A disintegrin and metalloproteinase with thrombospondin motifs 2 cleaves and inactivates Reelin in the postnatal cerebral cortex and hippocampus, but not in the cerebellum. Mol. Cell. Neurosci. 2019; 100:103401 31491533 10.1016/j.mcn.2019.103401

[53] Hu Y-S, Xin J, Hu Y, Analyzing the genes related to Alzheimer’s disease via a network and pathway-based approach. Alzheimers. Res. Ther. 2017; 9:29 28446202 10.1186/s13195-017-0252-z PMC5406904

[54] Grünblatt E, Riederer P. Aldehyde dehydrogenase (ALDH) in Alzheimer’s and Parkinson’s disease. J. Neural Transm. (Vienna) 2016; 123:83–90 25298080 10.1007/s00702-014-1320-1

[55] Miners J, van Hulle C, Ince S, Elevated CSF angiopoietin-2 correlates with blood-brain barrier leakiness and markers of neuronal injury in early Alzheimer’s disease. Res. Sq. 2023; 10.1038/s41398-023-02706-w PMC10770135 38182581

[56] Goyal MS, Blazey T, Metcalf NV, Brain aerobic glycolysis and resilience in Alzheimer disease. Proc. Natl. Acad. Sci. U. S. A. 2023; 120:e2212256120 36745794 10.1073/pnas.2212256120 PMC9963219

