# Supplementary material for: Predicting Alzheimer’s Cognitive Resilience Score: A Comparative Study of Machine Learning Models Using RNA-seq Data
Source: bioRxiv. 2024 Aug 26:2024.08.25.609610. Preprint. [Version 1] doi: 10.1101/2024.08.25.609610 (PMC11383294; doi:10.1101/2024.08.25.609610)
Supplement: 1 [file NIHPP2024.08.25.609610V1-supplement-1.pdf]

bioRxiv preprint doi: https://doi.org/10.1101/2024.08.25.609610; this version posted August 26, 2024. The copyright holder for this preprint (which was not certified by peer review) is the author/funder, who has granted bioRxiv a license to display the preprint in perpetuity. It is made available under aCC-BY 4.0 International license.

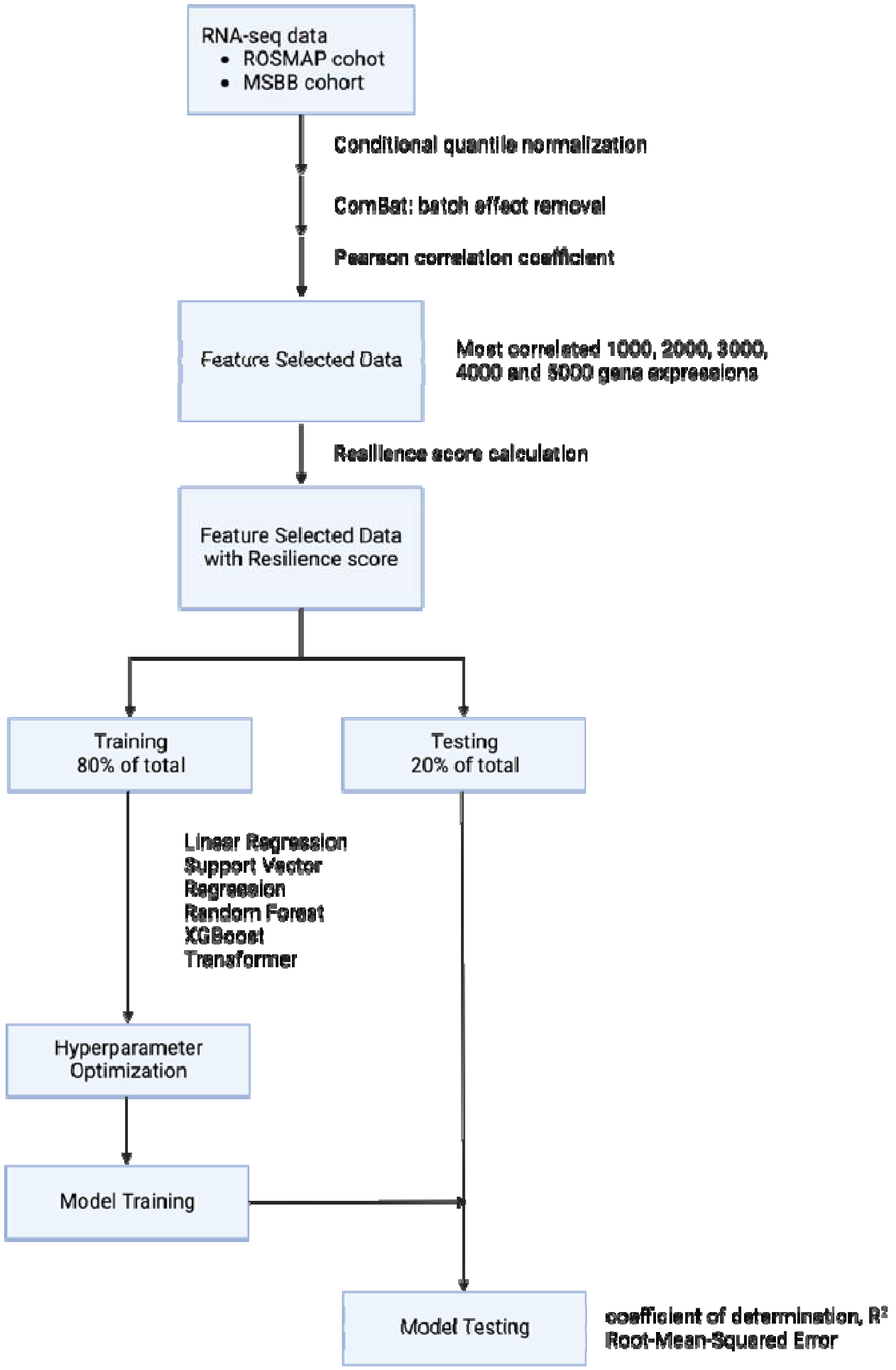

Supplementary Fig. S1. Flow chart of data acquisition, preprocessing, modeling, training, and testing. Conditional quantile normalization was performed on the data, and batch effects

bioRxiv preprint doi: https://doi.org/10.1101/2024.08.25.609610; this version posted August 26, 2024. The copyright holder for this preprint (which was not certified by peer review) is the author/funder, who has granted bioRxiv a license to display the preprint in perpetuity. It is made available under aCC-BY 4.0 International license.

were corrected using ComBat [42]. Genes with high Pearson correlation coefficients and resilience scores were selected for feature selection, with the resilience score being the target variable. The entire dataset was divided into training (80%) and testing (20%) datasets. Training and hyperparameter tuning were performed using the training dataset, and the accuracy of the model was evaluated using the testing dataset.

bioRxiv preprint doi: https://doi.org/10.1101/2024.08.25.609610; this version posted August 26, 2024. The copyright holder for this preprint (which was not certified by peer review) is the author/funder, who has granted bioRxiv a license to display the preprint in perpetuity. It is made available under aCC-BY 4.0 International license.

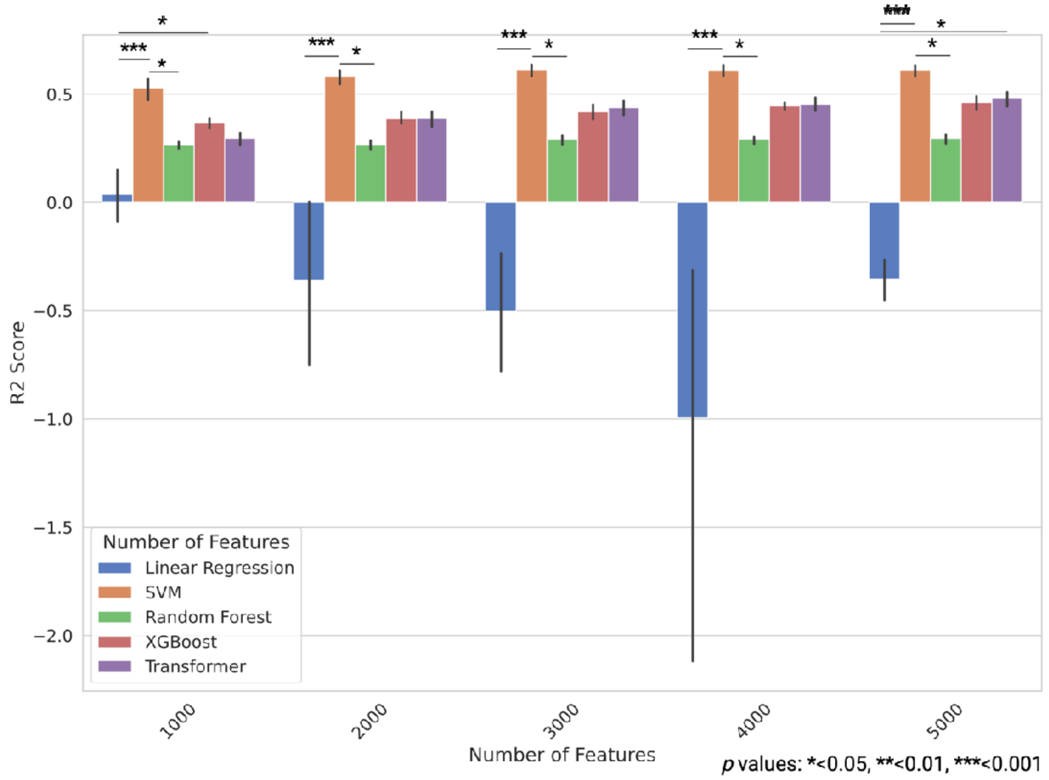

Supplementary Fig. S2. Prediction performances of different machine learning models based on R^2 values in the MSBB study data. The coefficient of determination (R^2) for each model is shown on the test data, with error bars representing the SE. Statistical significance was determined using the Kruskal–Wallis test followed by the Dunn's multiple comparison test, with p-values adjusted by Bonferroni correction (*<0.05, **<0.01, ***<0.001; n = 5 for 5-fold cross-validation).

bioRxiv preprint doi: https://doi.org/10.1101/2024.08.25.609610; this version posted August 26, 2024. The copyright holder for this preprint (which was not certified by peer review) is the author/funder, who has granted bioRxiv a license to display the preprint in perpetuity. It is made available under aCC-BY 4.0 International license.

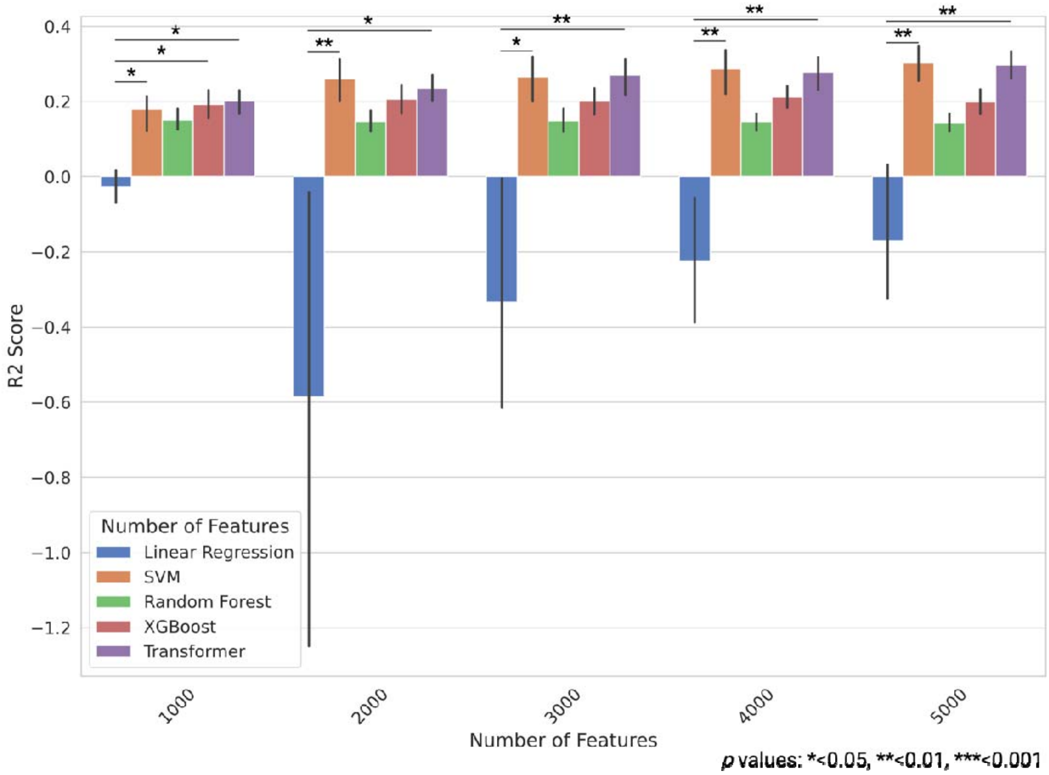

Supplementary Fig. S3. Prediction performances of various machine learning models based on R^2 values in the ROSMAP study data. The coefficient of determination (R^2) of each model is shown on the test data, with error bars representing the SE. Statistical significance was determined using the Kruskal–Wallis test followed by the Dunn's multiple comparison test, with p-values adjusted by Bonferroni correction (*<0.05, **<0.01, ***<0.001; n = 5 for 5-fold cross-validation).

bioRxiv preprint doi: https://doi.org/10.1101/2024.08.25.609610; this version posted August 26, 2024. The copyright holder for this preprint (which was not certified by peer review) is the author/funder, who has granted bioRxiv a license to display the preprint in perpetuity. It is made available under aCC-BY 4.0 International license.

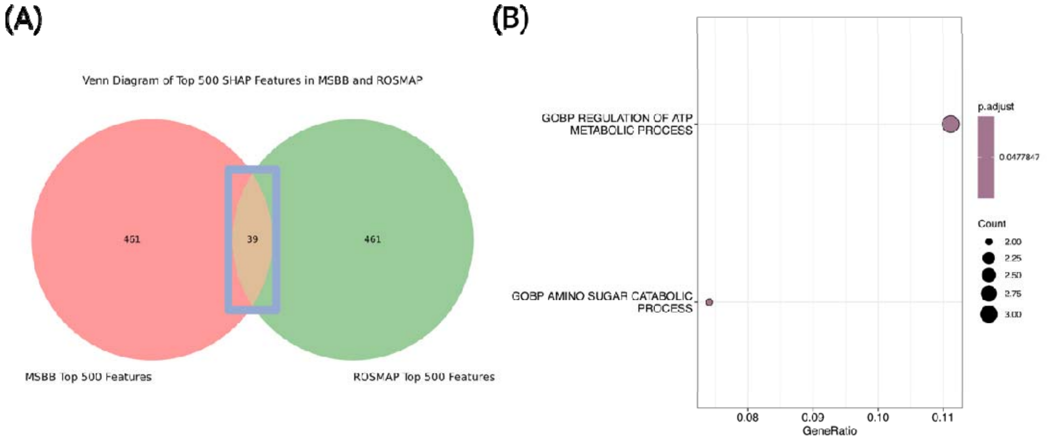

Supplementary Fig. S4. Enrichment analysis of top SHAP score genes. (A) Venn diagram showing the overlap between the top 500 features with the highest SHAP scores in the MSBB and ROSMAP datasets. An enrichment analysis was performed on the top 500 features from each dataset and the 39 genes common to both. (B) Gene ontology biological process (GOBP) pathways with an adjusted p-value (BH correction) of less than 0.05.
